# Functional Divergence in the Affinity and Stability of Non-Canonical Cysteines and Non-Canonical Disulfide Bonds: Insights from a VHH and VNAR Study

**DOI:** 10.3390/ijms25189801

**Published:** 2024-09-11

**Authors:** Mingce Xu, Zheng Zhao, Penghui Deng, Mengsi Sun, Cookson K. C. Chiu, Yujie Wu, Hao Wang, Yunchen Bi

**Affiliations:** 1 CAS and Shandong Province Key Laboratory of Experimental Marine Biology, Center for Ocean Mega-Science, Institute of Oceanology, Chinese Academy of Sciences, Qingdao 266071, China; 2 University of Chinese Academy of Sciences, Beijing 100049, China; 3 School of Data Science, University of Virginia, Charlottesville, VA 22904, USA; 4 Department of Biomedical Engineering, University of Virginia, Charlottesville, VA 22904, USA; 5 Shenzhen Bay Laboratory, Shenzhen 518055, China; 6 Laboratory for Marine Biology and Biotechnology, Qingdao Marine Science and Technology Center, Qingdao 266071, China

**Keywords:** single-domain antibodies, disulfide bonds, cysteines, nanobodies, intrabodies

## Abstract

Single-domain antibodies, including variable domains of the heavy chains of heavy chain-only antibodies (VHHs) from camelids and variable domains of immunoglobulin new antigen receptors (VNARs) from cartilaginous fish, show the therapeutic potential of targeting antigens in a cytosol reducing environment. A large proportion of single-domain antibodies contain non-canonical cysteines and corresponding non-canonical disulfide bonds situated on the protein surface, rendering them vulnerable to environmental factors. Research on non-canonical disulfide bonds has been limited, with a focus solely on VHHs and utilizing only cysteine mutations rather than the reducing agent treatment. In this study, we examined an anti-lysozyme VNAR and an anti-BC2-tag VHH, including their non-canonical disulfide bond reduced counterparts and non-canonical cysteine mutants. Both the affinity and stability of the VNARs and VHHs decreased in the non-canonical cysteine mutants, whereas the reduced-state samples exhibited decreased thermal stability, with their affinity remaining almost unchanged regardless of the presence of reducing agents. Molecular dynamics simulations suggested that the decrease in affinity of the mutants resulted from increased flexibility of the CDRs, the disappearance of non-canonical cysteine–antigen interactions, and the perturbation of other antigen-interacting residues caused by mutations. These findings highlight the significance of non-canonical cysteines for the affinity of single-domain antibodies and demonstrate that the mutation of non-canonical cysteines is not equivalent to the disruption of non-canonical disulfide bonds with a reducing agent when assessing the function of non-canonical disulfide bonds.

## 1. Introduction

**Figure 1:**
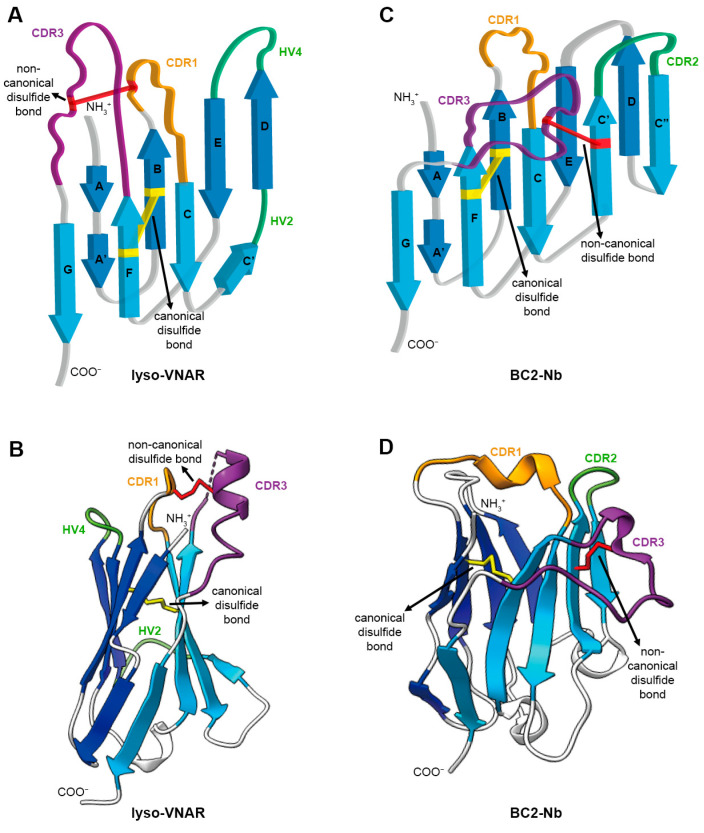
Architecture of single-domain antibody. (**A**) Topology diagram of anti-lysozyme VNAR (lyso-VNAR) with PDB ID:2I27. VNAR comprises a pair of antiparallel β sheets and antigen recognition region consisting of CDR and HV loops. The CDR and HV loops are colored in orange (CDR1), purple (CDR3), and green (HV2 and HV4). The canonical disulfide bond between β strand B and β strand F is colored in yellow. The non-canonical disulfide bond between CDR1 and CDR3 is colored in red. (**B**) The 3D structure of lyso-VNAR is colored as in (**A**). (**C**) Topology diagram of anti-BC2-tag VHH (BC2-Nb) with PDB ID:5IVO. Each component is colored the same as in (**A**), except for green, which shows the CDR2 in VHH instead of the HV loop in VNAR. (**D**) The 3D structure of BC2-Nb is colored as in (**C**).

Immunoglobulins naturally lacking light chains were found in camelids [1] and cartilaginous fish [2,3]. These antibodies, also referred to as heavy chain-only antibodies (HCAbs) or immunoglobulin new antigen receptors (IgNARs), possess variable domains with monomeric functionality, distinct from the paired heavy and light chain variable domains present in conventional IgGs [4]. These variable domains are designated as VHHs in camelids and VNARs in cartilaginous fish and are commonly referred to as single-domain antibodies (sdAbs). VHHs are also known as nanobodies (TM) [4]. Compared with classical IgGs, single-domain antibodies exhibit high solubility [5] and heat resistance and are stable under extreme pH conditions [6]. They have become valuable tools in biomedical research and medicine [7]. The ability of single-domain antibodies to recognize antigens is mainly provided by their complementarity determining regions (CDRs) and hypervariable loops (HVs) [8,9]. Despite the high variability of CDRs and HVs, the framework regions (FRs) of single-domain antibodies remain highly conserved at the species level. The cysteines at positions 23 and 104 (IMGT numbering system used throughout) forming conventional inter-β-sheet disulfide bonds are referred to as canonical cysteines. Non-canonical cysteines, however, were mainly located on CDR1-CDR3 or FR2-CDR3 [10]. Furthermore, a significant proportion of single-domain antibodies possess a non-canonical disulfide bond in addition to the conventional disulfide bond between FRs (Figure 1A–D) [11]. For VHHs, about 90% of VHHs derived from the Bactrian camel and dromedary have an extra cysteine in CDR1 or FR2, which enable the generation of a non-canonical disulfide bond which is predominantly situated between CDR1 and CDR3 or FR2 and CDR3. In alpacas, over 40% of VHHs contain a non-canonical cysteine in FR2, and the corresponding non-canonical disulfide bonds almost occur between FR2 and CDR3 [12]. Shark VNARs contains a considerably higher proportion of non-canonical disulfide bonds than alpaca VHHs [13,14]. For instance, over 70% of the identified VNARs in white-spotted bamboo sharks feature one non-canonical disulfide bond between CDR1 and CDR3 [14]. Non-canonical disulfide bonds are also prevalent in single-domain antibodies from other shark species, including the nurse shark (*Ginglymostoma cirratum*) [13], spiny dogfish (*Squalus acanthias*) [15], and banded hound shark (*Triakis scyllium*) [16].

The reasons for the high prevalence of non-canonical disulfide bonds in single-domain antibodies remain unclear. Furthermore, the precise function of these non-canonical disulfide bonds has not been fully elucidated, although a few studies suggest that naturally occurring non-canonical cysteines contribute to the stability, function, and diversity of single-domain antibodies [17,18]. To our knowledge, previous studies have explored the function of non-canonical disulfide bonds using the cysteine mutation method. However, it should be noted that this method cannot truly replace disulfide bond breakage in a reducing environment. Given the therapeutic potential of single-domain antibodies, especially their ability to target intracellular molecules through cell permeability [19], the performance of single-domain antibodies in cytosol-reducing environments is of particular concern. Thus, it is crucial to assess the changes in affinity and stability of single-domain antibodies under reducing conditions and compare these changes with those produced by mutations.

In this study, we selected a VNAR targeting lysozyme and a VHH targeting the 12 amino acid BC2 peptide and investigated the impact of reducing agent treatment or cysteine mutations on their affinity and stability. Our results indicate a significant decrease in affinity in the mutants but not in the samples treated with reducing agents. We further observed this phenomenon in another VHH and VNAR sample. Molecular dynamics (MD) simulations suggest that the change in affinity may be attributed to the increased flexibility of the CDRs, as well as the disappearance or perturbation of direct and indirect interactions between the non-canonical cysteines and antigen. In terms of stability, both the mutants and reduced-state samples of single-domain antibodies displayed similar decreases in thermal stability. This is consistent with recent antibody engineering studies [20,21]. Kim et al. indicated that integrating non-canonical disulfide bonds into Ig V domain FRs can enhance the stability of these domains while preserving antigen binding [22]. However, it is important to note: unlike the introduced non-canonical disulfide bonds in antibody engineering, our study focuses on naturally occurring non-canonical disulfide bonds. The term “non-canonical disulfide bonds” specifically denotes naturally occurring instances throughout the manuscript unless otherwise stated.

## 2. Results

### 2.1. Non-Canonical Cysteine Residues but Not Non-Canonical Disulfide Bonds Contribute to Binding Affinity

The single-domain antibody lyso-VNAR, derived from nurse sharks, has been shown to inhibit lysozyme activity and contain two disulfide bonds. The canonical disulfide bond resides within the hydrophobic core of the protein, while the non-canonical disulfide bond, formed between Cys30 in CDR1 and Cys112.1 in CDR3 (IMGT numbering system), is located on the protein surface and sensitive to environmental changes. To comprehensively assess the impact of non-canonical cysteine mutations and identify lyso-VNAR mutants with relatively higher binding affinities, a site saturation mutagenesis library of all possible combinations of 20 different amino acids at Cys30 and Cys112.1 was constructed. Following two rounds of panning and phage ELISA, judging by their absorbance values, the top 40 positive clones were included in the following research. Of these, 30 retained the original lyso-VNAR sequence with no mutations. The remaining 10 clones were found to contain eight distinct mutants: lyso-VNAR_C30P_C112.1T_, lyso-VNAR_C30L_C112.1L_, lyso-VNAR_C30W_C112.1V_, lyso-VNAR_C30L_C112.1V_, lyso-VNAR_C30A_C112.1S_, lyso-VNAR_C30G_C112.1A_, lyso-VNAR_C30L_C112.1A_, and lyso-VNAR_C30G_C112.1L_. The mutants lyso-VNAR_C30G_C112.1A_ and lyso-VNAR_C30G_C112.1L_ appeared twice each in 10 clones.

**Figure 2:**
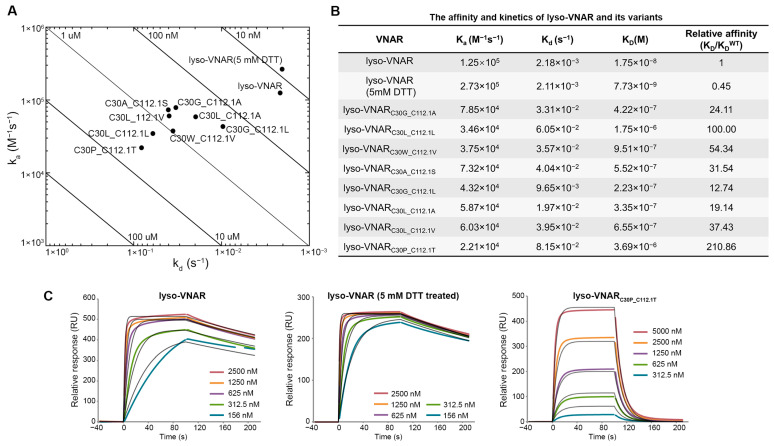
Binding experiments between lyso-VNAR and lysozyme. (**A**) Rate plane with Isoaffinity Diagonals plot for lyso-VNAR wild type, reduced-state sample, and eight mutants. (**B**) Equilibrium and kinetic parameters obtained from SPR. (**C**) Analysis by SPR spectra of the binding of lyso-VNAR in the wild type, reduced-state, and exemplary mutant lyso-VNAR_C30P_C112.1T_ to lysozyme. Different concentrations of VNAR were flowed over a cell immobilized with lysozyme. The figure shows the concentration-dependent binding response and 1:1 fitting model.

The binding kinetics of the eight mutants, lyso-VNAR, and DTT-treated lyso-VNAR were analyzed via surface plasmon resonance (SPR) to determine their kinetic association rate constants (k_a_), kinetic dissociation rate constants (k_d_), and equilibrium dissociation constants (K_D_) for lysozyme (Figure 2A–C). In the wild-type and DTT-treated samples, iodoacetamide was used to alkylate the reduced sulfhydryl group, and then the presence and removal of the non-canonical disulfide bonds was detected with mass spectrometry. The results indicate that only the non-canonical disulfide bond was broken in the presence of DTT (Figure S1). The K_D_ of lyso-VNAR was measured to be 17.5 nM. The mutants displayed K_D_ values ranging from 223 nM to 3.69 μM, representing a 13–211 fold increase relative to lyso-VNAR. This increase in K_D_ resulted from a 2–6 fold decrease in k_a_ and a 4–37 fold increase in k_d_ (Figure 2B and Figure S2A–H). The reduced-state lyso-VNAR exhibited a K_D_ value of 7.73 nM, which was found to be comparable to that of native lyso-VNAR. These findings suggest that the non-canonical disulfide bond in lyso-VNAR may not play a crucial role in the lysozyme recognition process.

**Figure 3:**
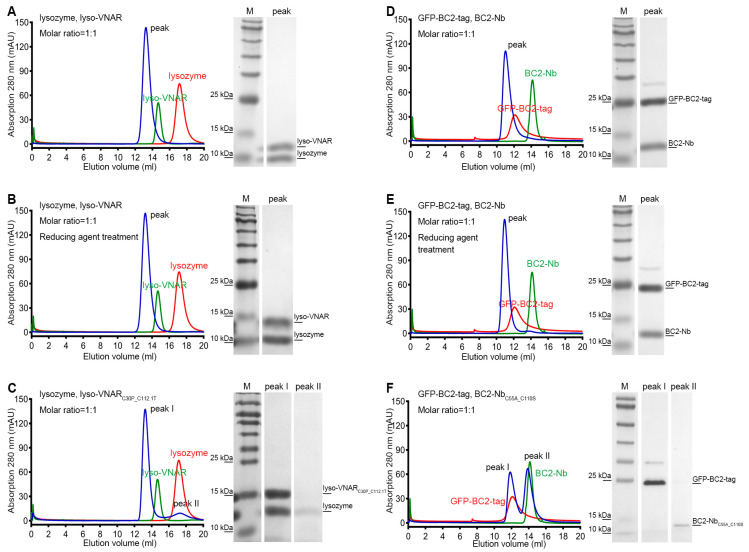
Size exclusion chromatography and SDS-PAGE analysis of the single-domain antibody and antigen complex formation. Single-domain antibodies were incubated with the corresponding antigen at a 1:1 molar ratio and then loaded for size exclusion chromatography followed by SDS-PAGE analysis. The lyso-VNAR and BC2-Nb samples, regardless of the disulfide bond state, were found to contain only one peak, and the SDS-PAGE analysis verified the single-domain antibody-antigen complex’s formation (**A**,**B**,**D**,**E**). There were two elution peaks for the lyso-VNAR_C30P___C112.1T_. The first and secondary peaks were diagnosed as the complex and lysozyme through SDS-PAGE analysis, respectively (**C**). Size exclusion chromatogram showing two peaks appearing from the elution profile of BC2-Nb_C55A___C110S_. The first and secondary peaks correspond to GFP-BC2-tag and BC2-Nb_C55A___C110S_, respectively (**F**). The red and green curves represent individual SEC profiles of the corresponding antigen and antibody, respectively, providing a clearer depiction of the shifts in elution positions of the complex peaks.

To validate the differences in binding affinity observed among lyso-VNAR, reduced-state lyso-VNAR, and the mutants, co-purification experiments were performed using size exclusion chromatography (SEC). Lyso-VNAR and its mutants with a fused purification tag had a total molecular weight of 13.3 kDa and were eluted at 14.2 mL from a Superdex^TM^ 75 10/300 GL column (Cytiva, Uppsala, Sweden). The lysozyme was a 14.3 kDa protein, eluting at a volume of 16.3 mL from a Superdex^TM^ 75 10/300 GL column. When lyso-VNAR was co-purified with lysozyme, a single peak was eluted at 13.2 mL, and SDS-PAGE analysis identified the formation of a lyso-VNAR-lysozyme complex (Figure 3A). The reduced counterpart yielded similar results, with a single elution peak at an elution volume of 13.2 mL, indicating the interaction between lyso-VNAR and lysozyme was unaffected in a reducing environment only with breakage of the disulfide bond (Figure 3B). However, lyso-VNAR_C30P_C112.1T_, which had the highest binding affinity value among the eight mutants, showed two peaks when incubated with lysozyme at a 1:1 molar ratio. Other than the complex peak at 13.2 mL, the additional peak overlapped with the SEC profiles of the antigen and was confirmed to be lysozyme through SDS-PAGE analysis (Figure 3C). These findings suggest that the non-canonical disulfide bond breakage in a reducing environment does not affect the binding of lyso-VNAR and lysozyme. Rather, the double-cysteine mutation in the mutants results in a weaker binding capacity and dissociation of the complex, consistent with the results from the SPR experiment.

**Figure 4:**
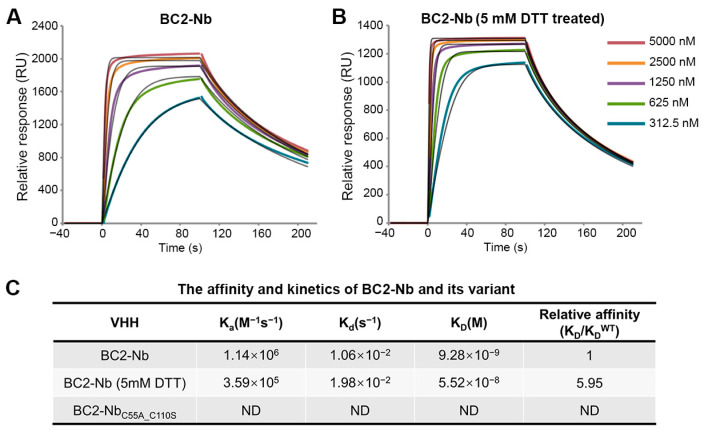
SPR experiments for binding affinity determination between BC2-Nb and GFP-BC2-tag. The GFP-BC2-tag was stably immobilized to the sensor chip. SPR sensorgrams were obtained for the injections of different concentrations of BC2-Nb and BC2-Nb reduced-state samples (**A**,**B**). The 1:1 fitting model curves are also shown in the corresponding pictures. Equilibrium and kinetic parameters calculated from SPR experiments. The binding between BC2-Nb_C55A___C110S_ and GFP-BC2-tag was too weak to detect (**C**).

An anti-BC2-tag VHH from camelids was also included in this study for comparison. The BC2-tag is a 12 amino acid peptide which is commonly used as a protein tag for affinity chromatography [23]. BC2-Nb was generated through llama immunization, had nM-level binding affinity for the BC2-tag, and featured a canonical and non-canonical disulfide bond. Our SPR analysis revealed that the capability of BC2-Nb binding with the BC2-tag was nearly equivalent, regardless of the presence of a reducing agent (Figure 4A,B). Conversely, BC2-Nb_C55A_C110S_ was identified as a representative mutant in a previous study on BC2-Nb. This mutant exhibited non-canonical cysteine mutations and the absence of a non-canonical disulfide bond. It showed undetectable binding to the BC2-tag (Figure 4C), which is consistent with a previous report [24].

Additionally, we conducted SEC-based co-purification experiments to examine the interactions between BC2-Nb and BC2-tag. The BC2-tag was fused with green fluorescent protein (GFP) at its N-terminal to facilitate purification, resulting in a molecular weight of 31.1 kDa and an elution volume of 12.1 mL from a Superdex^TM^ 75 10/300 GL column. BC2-Nb and its mutant had a molecular weight of 14.7 kDa and an elution volume of 14.3 mL from the same column. After incubating BC2-Nb and GFP-BC2-tag at a 1:1 molar ratio, a single peak eluting at 11.0 mL was obtained, and the SDS-PAGE results indicated that a stable complex formed (Figure 3D). The reducing agent treatment sample showed similar results to its non-reducing counterpart (Figure 3E). However, the BC2-Nb_C55A_C110S_ sample revealed distinct results, with two peaks appearing after incubation and co-purification. The first peak was located at 11.8 mL, which was consistent with the SEC profiles of GFP-BC2-tag, while the second peak was located at 13.9 mL, which overlapped with the chromatography of BC2-Nb_C55A_C110S_ purification alone. The SDS-PAGE results further confirmed that the two peaks corresponded to GFP-BC2-tag and BC2-Nb_C55A_C110S_ (Figure 3F). In summary, disrupting the non-canonical disulfide bond of BC2-Nb in a reducing environment did not significantly impact its binding to BC2-tag, whereas the double-cysteine mutation BC2-Nb_C55A_C110S_ resulted in a complete loss of binding.

The importance of non-canonical cysteines in antigen binding was further inspected through two additional single-domain antibody studies through SEC-based co-purification experiments. The anti-human serum albumin (HSA) VNAR, termed B8-VNAR, was screened from a shark-derived immune VNAR library in our lab and contained a non-canonical disulfide bond bridging CDR1 and CDR3. After incubating B8-VNAR with HSA, only one elution peak appeared regardless of the presence or absence of a reducing agent (Figure S3A,B), while the non-canonical cysteine mutation B8-VNAR_C37A_C109S_ sample showed an additional VNAR peak (Figure S3C). These co-purification results indicate that the non-canonical disulfide bond in B8-VANR did not affect the affinity of single-domain antibodies, but mutation of the non-canonical cysteines led to decreased binding of B8-VNAR to HSA.

M2 ectodomain (M2e) peptide contains 23 amino acids and is highly conserved among different influenza A virus subtypes. The M2e peptide was fused with the MBP tag and (G_4_S)_2_ linker sequentially at its N-terminal to increase the expression yield. M2e-Nb was isolated from the llama and had a non-canonical disulfide bond between FR2 and CDR3 (Figure S4A) [25]. The wild type and reducing agent-treated sample exhibited similar behavior in the SEC co-purification experiment. Both of them exhibited three peaks. The first two peaks, preceding the individual antigen SEC peak, correspond to the antigen-single-domain antibody complex, and the third tiny peak was identified as the M2e-Nb (Figure S3D,E). The performance of M2e-Nb_C55A_C112.1S_ was completely different compared with those of the wild type and reduced samples, with peaks representing the antigen and antibody without complex elution (Figure S3F). Combining these results shows that there was no significant difference in M2e-Nb binding to M2e regardless of the presence or absence of the reducing agent, while the non-canonical cysteine mutant M2e-Nb_C55A_C112.1S_ lost its binding capacity with M2e.

### 2.2. Dynamical and Interactional Characteristics of the Non-Canonical Cysteines

A 1600 ns MD simulation experiment was performed to study the dynamics of the single-domain antibodies [26]. Overall, the single-domain antibodies remained stable, with the RMSD of all systems increasing for approximately 50 ns before reaching stability (Figure S5A). Residues 88–104 in lyso-VNAR and residues 102–113 in BC2-Nb exhibited considerable fluctuation corresponding to the CDR3 of each single-domain antibody (Figure S5B). This result indicates that CDR3 is flexible and important for antigen binding.

**Figure 5:**
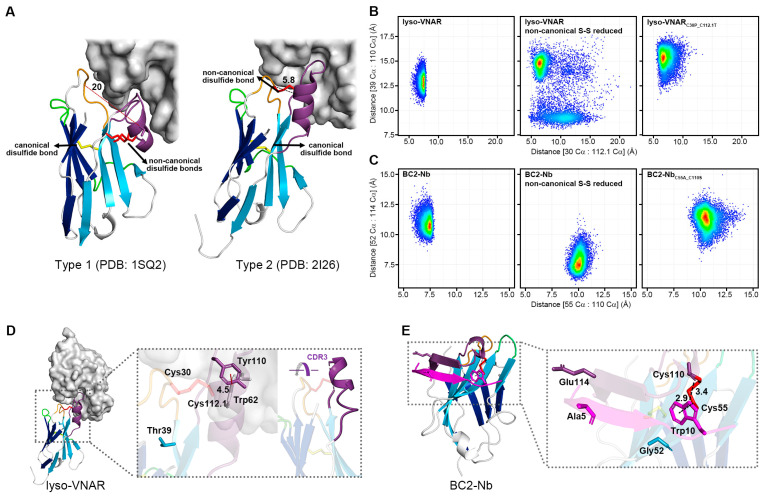
Density scatter plot of distributions of specific Cα-Cα distances for different systems and the structure of the single-domain antibody in a complex with the antigen. (**A**) Type 1 VNAR against lysozyme and Type 2 lyso-VNAR recognized the same crack-like epitope but bound differently. CDR1 (orange) and CDR3 (purple) of the Type 1 VNAR against lysozyme fastened at the edge, while the Type 2 lyso-VNAR bound to the inside of the cleft through CDR1 (orange) and CDR3 (purple). Accordingly, the distance between CDR1 and CDR3 in the Type 2 lyso-VNAR (Cys30-Cys112.1, 5.8 Å) was much smaller than that of the Type 1 VNAR against lysozyme (Ala31-Ser113, 20 Å). The density scatter plot shows the distributions of the distances between the Cα of residue 30 and the Cα of residue 112.1, between the Cα of Thr39 and the Cα of Tyr110 in the lyso-VNAR wild type, reduced state, and lyso-VNAR_C30P___C112.1T_ (**B**), between the Cα of residue 55 and the Cα of residue 110, and between the Cα of Gly52 and the Cα of Glu114 in the BC2-Nb wild type, reduced state, and BC2-Nb_C55A___C110S_ (**C**). Density visualized using a color gradient from red to blue, indicating a density decrease from high to low, respectively. (**D**) The structure of lyso-VNAR bound to lysozyme. The lyso-VNAR is shown as the cartoon, and lysozyme is shown as the surface. The panel presents the relative positions of the residues involved in the MD simulations and the T-shaped π–π interaction between Trp62 in the lysozyme and Tyr110 in the lyso-VNAR, with a distance of 4.5 Å between the centers of the aromatic rings. (**E**) The structure of BC2-Nb bound to BC2-tag. The BC2-tag is colored in magenta, and the CDRs are colored as in Figure 1. The panel presents the relative positions of the residues involved in the MD simulations and the primary intermolecular interaction residues of the BC2-tag (Ala5 and Trp10). Trp10 in the BC2-tag interacted with Cys55 of the BC2-Nb through a CH–π interaction with a distance of 2.9 Å and formed a hydrogen bond with Cys110 of the BC2-Nb with a distance of 3.4 Å.

Based on the location and number of non-canonical cysteine residues, the VNAR could be classified into several groups (e.g., Type 1 and Type 2 for *Ginglymostoma cirratum* (Figure 5A)). Both the Type 1 VNAR against lysozyme and Type 2 lyso-VNAR targeted the same grooved epitope encompassing a large part of the substrate-binding pocket of lysozyme, a known binding site for anti-lysozyme VHHs as well [27,28,29,30,31]. However, their binding modes differed; CDR3 in the Type 1 VNAR against lysozyme bent over to clasp the extruded lip area with CDR1, while CDR1-CDR3 in Type 2 lyso-VNAR stretched out to bind to the inside of the crack (Figure 5A). The distance between CDR1 and CDR3 in the Type 2 lyso-VNAR (Cys30-Cys112.1) mainly stayed within 5.8–7.5 Å during the 1600 ns MD simulation for all samples, including lyso-VNAR, the reduced-state sample, and the mutant (Figure 5B). This short distance enabled the Type 2 lyso-VNAR to bind to the recessed epitope and form a complex, which is consistent with the results of the SEC-based co-purification experiments. Neither removal of the disulfide bonds nor cysteine mutation could convert a Type 2 lyso-VNAR to adopt the Type 1 VNAR conformation when complexed with lysozyme.

The MD simulations also calculated the distance between Thr39 and Tyr110 in the Type 2 lyso-VNAR. Thr39 resides in the stable β strand of the frame, while Tyr110 is in the flexible CDR3 loop and interacts with Trp62 of the lysozyme via a T-shaped π–π stacking interaction with a distance of 4.5 Å between the centers of the aromatic rings (Figure 5D). The size exclusion chromatography co-purification experiment revealed that lyso-VNAR_Y110A_ mutation diminished the binding affinity to lysozyme (Figure S5C), similar to lyso-VNAR_C30P_C112.1T_. In an anti-lysozyme VHH which recognizes similar epitopes, a tyrosine (Y111 from PDB ID: 6JB8) is also present at a position similar to Tyr110, and it is vital to antigen-antibody binding. After mutation from tyrosine to alanine, the affinity decreased by over 2000 fold [31].

During the 1600 ns equilibrium simulation, the distance between Thr39 and Tyr110 in the wild-type system was approximately 12.5–13.5 Å, but it increased to 14.8 Å in the reduced-state system and to approximately 15.5 Å in the mutated system (Figure 5B and Figure S6A). This increased distance may not be due to CDR3 (where Tyr110 is located) moving toward or away from CDR1 but rather lateral sliding parallel to CDR1 (Figure 5D). Therefore, we propose that non-canonical cysteine mutations might induce lateral sliding of CDR3, potentially impairing Tyr110’s binding to the epitope and ultimately causing the observed affinity alterations. This could represent a model for understanding how non-canonical cysteines impact the affinity of single-domain antibodies by influencing other epitope-binding amino acids, even without direct antigen contact.

Trp10 in the BC2-tag, located in the hydrophobic pocket, interacted with Cys55 of the BC2-Nb through a CH–π interaction and formed a hydrogen bond with Cys110 of the BC2-Nb. In the wild-type system, the distance between the Cα of Cys55 and the Cα of Cys110 was approximately 7.5 Å, which increased to 10 Å and 10.5 Å in the reduced-state and mutated systems, respectively (Figure 5C and Figure S6B). The Cys55 was located at the stable β strand of the frame, implying limited positional fluctuations. Thus, the increased distance may have arisen from the flexibility of Cys110, which was located at the flexible CDR3 loop. In the reduced-state system, the excessive distance precluded the hydrogen bond between Cys110 and Trp10, although the stable Cys55 could still interact with Trp10 via the CH–π interaction, contributing to the binding affinity between the BC2-Nb and BC2-tag. The mutant BC2-Nb_C55A_C110S_ may have partially disrupted the hydrophobic pocket, which is essential for interaction with Trp10. Furthermore, variation in the interaction was observed in the BC2-Nb binding with the middle part of the BC2-tag. Upon binding, part of the CDR3 loop in the BC2-Nb formed an antiparallel β sheet with the BC2-tag, sandwiching it between the two β strands where Gly52 and Glu114 were located (Figure 5E). As Gly52 and Glu114 were involved in the interaction with the middle part of the BC2-tag, the distance between these two residues was calculated to evaluate the binding capability to this region, which contained three residues (Ala5, Ala6, and Val7 of the BC2-tag) with small side chains. According to the crystal structure of the complex (PDB ID: 5IVN), the middle part of the BC2-tag had limited interactions with the BC2-Nb, particularly Ala5, which lacked any contact with its antibody. The distance between Gly52 and Glu114 in the reduced-state system was 7.5 Å, much shorter than 10.5 Å in the wild-type system and 11 Å in the mutated system (Figure 5C). The shortened distance may have enhanced the interaction with amino acids with smaller side chains in the BC2-tag, such as Ala5, which was consistent with previous reports that the mutation of Ala5 to Arg5 can increase binding affinity [32,33].

### 2.3. Influences of Non-Canonical Disulfide Bonds on the Stability of VHHs and VNARs

The shark IgNAR is exceptionally stable, probably due to the selective pressure brought on by the high concentration of urea in shark blood [34]. At the molecular level, the presence of non-canonical disulfide bonds may increase stability, as the disulfide bond is broadly used in protein engineering to boost protein stability [35,36]. The lyso-VNAR and BC2-Nb under reducing and non-reducing conditions, as well as their mutants, were treated with an increase in temperature to measure the melting temperature (Tm) at which 50% of the protein underwent unfolding (Figure S7A–M). The Tm values for the lyso-VNAR were 56.4 ± 0.8 °C. The mutants and reduced-state sample of lyso-VNAR showed decreased Tm values. The Tm of the lyso-VNAR mutants dropped by about 9–19 °C, while the Tm of the reduced-state lyso-VNAR dropped by approximately 15 °C (Table 1). For the BC2-Nb, a lower Tm was also observed for both reduced-state sample and mutant version, relative to the wild type (Table 2). These results indicate that disruption of the non-canonical disulfide bonds via mutation or reduction could decrease thermal stability.

## 3. Discussion

The role of non-canonical disulfide bonds in VHHs and VNARs is crucial, as they are situated on the surface of single-domain antibodies and primarily involved in CDR loops, which are essential for antigen binding. Non-canonical disulfide bonds and cysteines are predominantly located in the CDR1-CDR3 or FR2-CDR3 regions. In VHHs containing non-canonical disulfide bonds from camels, including both dromedaries and Bactrian camels, nearly 100% of the first non-canonical cysteines are found in CDR1, whereas in alpacas, over 95% are found in FR2 [12]. Regarding VNARs, approximately 79% of those identified in a bamboo shark immunized library and 57% of those in a nurse shark naive library exhibit CDR1-CDR3 disulfide bonds [13,14]. This prevalence of non-canonical disulfide bonds in restricted positions could be attributed to the germline encoding of cysteine residues in CDR1 or FR2 [12,18].

Mutational analysis was conducted on a total of six VHHs to assess the function of the non-canonical disulfide bonds between CDR1 and CDR3, a previously discussed representative position. Some mutants exhibited a significant decrease in affinity [17,18]. However, it is unclear whether this affinity change was due to cysteine residue mutations or disulfide bond breakage. To elucidate this, we employed non-canonical cysteine mutagenesis as well as DTT treatment experiments. Surprisingly, the disruption of non-canonical disulfide bonds after DTT treatment had minimal impact on the binding affinity, contrary to both our observations and the reported data for non-canonical cysteine mutants. This suggests the importance of non-canonical cysteine residues rather than non-canonical disulfide bonds in antigen binding. Crystal structure analysis evidenced the non-canonical cysteine’s direct interaction with antigens. Pellis et al. utilized a bacterial two-hybrid system to select intracellular functional nanobodies, with a notable proportion of these binders showing competitive affinity featuring cysteine pairs in their CDR1 and CDR3 loops. In particular, two binders consistently performed well in both oxidizing (extracellular, phage display system) and reducing (intracellular, bacterial two-hybrid system) environments [37]. These instances closely resemble our research findings, and our results provide a plausible hypothesis for this phenomenon. It should be pointed out that non-canonical cysteines do not necessarily participate in antigen binding, in which case the cysteine mutation will not change the affinity between an antigen and a single-domain antibody [17,18]. In terms of stability, cysteine mutations and breakage of non-canonical disulfide bonds have both been shown to decrease the thermal stability of lyso-VNAR and BC2-Nb.

In conclusion, this study complements previous research and shows that non-canonical cysteines rather than non-canonical disulfide bonds have an impact on the binding affinity when cysteines are involved in antigen recognition. In such cases where disulfide bonds are being studied, it is worth noting that mutation and the reducing agent treatment should not be considered interchangeable. Because mutation may not accurately replicate the disulfide bond breakage which occurs in a reducing environment, equal interpretation of these two experimental approaches may result in inconclusive findings. Single-domain antibody-derived therapies with a promising preclinical effect are expected to bolster the rise of intrabody therapy, which could exert biological activities in a specific intracellular compartment, such as a cytosol-reducing environment. This work demonstrates that it is unnecessary to exclude intrabodies which contain non-canonical disulfide bonds during the early screening in order to guarantee functionality in application under reducing conditions. Therefore, it is crucial to recognize the significance of non-canonical cysteines in these cases, rather than solely focusing on non-canonical disulfide bonds.

## 4. Materials and Methods

### 4.1. Construction of the Cys-Cys Saturation Mutagenesis Library

The non-canonical “Cys” codon within the CDR1 and CDR3 of lyso-VNAR was subjected to PCR-mediated randomization using degenerate primers. These primers contained an “NNN” sequence which replaced the “TGY” codon. The PCR products were purified using a gel extraction kit (Qiagen, Hilden, Germany) and then ligated into the pComb3XSS vector (Addgene, Watertown, MA, USA) after being digested with the restriction enzyme SfiI (NEB, Ipswich, MA, USA). The ligation products were then electroporation transformed into freshly prepared *E. coli* ER2738 (NEB, Ipswich, MA, USA) and plated on 2YT agar plates containing ampicillin. The library size of the lyso-VNAR with randomized Cys was calculated to be 10^5^. The clones were incubated overnight and stored at −80 °C in a glycerol solution with a 25% final concentration in 2YT medium.

### 4.2. Phage Display and Phage ELISA

The in vitro selection of the Cys-Cys saturation mutagenesis lyso-VNAR library against lysozyme was performed similarly according to the procedure described previously by Pardon et al. [38]. Before panning, the lysozyme was diluted to 10 μg/mL or 5 μg/mL in PBS buffer (137 mM NaCl, 2.7 mM KCl, 10 mM Na_2_HPO_4_, and 1.8 mM KH_2_PO_4_ (pH: 7.5)) and then coated onto 96 well plates (Corning, New York, NY, USA) at a volume of 50 μL per well and incubated at 4 °C overnight. The plates were then blocked with 3% milk powder in PBS buffer (pH: 7.5) for 2 h at room temperature. The library cells were inoculated into 50 mL of 2YT medium and cultured until the OD_600_ reached 0.6. At this point, helper phage M13KO7 (NEB, Ipswich, MA, USA) was added and incubated for 2 h at room temperature on a vibrating shaker. The uninfected phage was removed by centrifugation at 4000 rpm for 10 min, and the pellet was resuspended for culturing. After culturing overnight, the bacterial pellet was discarded by centrifugation at 8000 rpm for 10 min and the supernatant was collected. Then, the phage was precipitated by centrifugation at 8000 rpm for 10 min with a buffer containing 20% (*v*/*v*) PEG6000 and 2.5 M NaCl. The collected phage was then added to the 96 well plate at a volume of 100 μL per well and incubated for 2 h at RT. After washing with 250 μL 0.05% PBST 15 times and eluting with 100 μL 0.2 M Gly-HCl (pH: 2.2) for 15 min, the eluent was neutralized with 15 μL 1 M Tris-HCl (pH: 10.0), and the target-specific recombinant phage was collected. After two rounds of panning (with lysozyme of 10 μg/mL or 5 μg/mL coated), 80 individual clones were randomly picked and screened via phage ELISA. The phage supernatant was added to the 96 well plates coated with 5 μg/mL lysozyme at a volume of 100 μL per well, and binding was detected using HRP-conjugated mouse anti-M13 antibody (Sino Biological, Beijing, China). The absorption at 450 nm and 630 nm was measured after adding the TMB substrate solution (TIANGEN, Hilden, Germany), and the genes of the top 40 ranking clones which tested positive were sequenced.

### 4.3. Antigen Expression and Purification

The lysozyme from egg (Sigma, Darmstadt, Germany) and human serum albumin (Beijing Solarbio Science & Technology Co., Ltd., Beijing, China) were dissolved in PBS buffer (pH: 7.5) and purified using size exclusion chromatography with a Superdex^TM^ 75 10/300 GL column (Cytiva, Uppsala, Sweden) separately. The GFP gene (GENEBANK: AF242364.2) was fused to the N-terminal of the BC2-tag and then cloned into a pET28a vector (Novagen, Beijing, China), which was then transformed into *E. coli* BL21(DE3) cells (Tsingke Biotechnology Co., Ltd., Beijing, China). The gene of the M2e peptide with a (G_4_S)_2_ linker was recombined into the pET28a-MBP vector (Novagen, Beijing, China) and then transformed into *E. coli* BL21(DE3) cells (Tsingke Biotechnology Co., Ltd., Beijing, China). The cells were cultured in LB medium supplemented with 50 mg/L kanamycin at 37 °C, and when the OD_600_ reached 0.6, the antigen was induced for expression with 0.1 g/L Isopropyl β-D-1-thiogalactopyranoside (IPTG) for 18 h at 28 °C for the GFP-BC2-tag and 16 °C for the MBP-M2e. The cells were harvested by centrifugation at 8000 rpm for 5 min and resuspended in PBS buffer (pH: 7.5). The cell suspension was lysed using a high-pressure homogenizer (Union-Biotech (shanghai) Co., Ltd., Shanghai, China), and the insoluble material was removed by centrifugation at 14,000 rpm for 30 min. The supernatant was batch-incubated with Ni-NTA agarose (Qiagen, Hilden, Germany) at 4 °C for 60 min, followed by Ni-NTA affinity chromatography. The resin was washed with 50 mL of WB1 buffer containing PBS, 1 M NaCl, and 20 mM imidazole (pH: 7.5) and 50 mL of WB2 buffer containing PBS, 0.6 M NaCl, and 40 mM imidazole (pH: 7.5), and the protein was eluted with EB buffer containing PBS and 300 mM imidazole (pH: 7.5). The antigen was further purified using size exclusion chromatography with a Superdex^TM^ 75 10/300 GL column (Cytiva, Uppsala, Sweden) in PBS buffer (pH: 7.5).

### 4.4. Single-Domain Antibody Expression and Purification

The amino acid sequences of the lyso-VNAR, BC2-Nb, and M2e-Nb were obtained from the Protein Data Bank with codes 2I27, 5IVO, and 6S0Y, respectively (Figure S4A,B). The genes encoding the aforementioned single-domain antibodies were synthesized (Tsingke Biotechnology Co., Ltd., Beijing, China), and their mutants were generated by site mutagenesis [39]. All of the coding sequences, except for M2e-Nb_C55A_C112.1S_, were subcloned into the pET22b vector (Novagen, Beijing, China) and transformed into *E. coli* BL21(DE3) cells (Tsingke Biotechnology Co., Ltd., Beijing, China). The cells were grown in 2YT medium containing 100 mg/L ampicillin at 37 °C. Once the OD_600_ reached 0.6, single-domain antibodies were induced for expression in the periplasm by adding IPTG with a final concentration of 0.1 g/L. After expression for 18 h at 28 °C for the lyso-VNAR, lyso-VNAR mutants, BC2-Nb, BC2-Nb_C55A_C110S_, and B8-VNAR_C37A_C109S_ and 16 °C for the M2e-Nb and B8-VNAR, the periplasmic fractions were obtained via the osmotic shock method [40]. Briefly, the bacteria were collected by centrifugation at 5000 rpm for 15 min. The cell pellet of 1 L culture was sequentially resuspended and incubated for 1 h at 4 °C in each of the following buffers: 20 mL of ice-cold TES buffer containing 0.2 M Tris, 0.57 mM EDTA, 0.5 M sucrose (pH: 8.0), and 40 mL of ice-cold TES/4 buffer. The suspension was then centrifuged for 30 min at 14,000 rpm, and the supernatant was recovered as the periplasmic extract. The periplasmic extract was batch-incubated with Ni-NTA agarose (Qiagen, Hilden, Germany) at 4 °C for 60 min. The resin was washed with 50 mL of WB1 buffer containing PBS, 1 M NaCl, and 20 mM imidazole (pH: 7.5) and 50 mL of WB2 buffer containing PBS, 0.6 M NaCl, and 40 mM imidazole (pH: 7.5). The single-domain antibodies were then eluted with EB buffer containing PBS and 300 mM imidazole (pH: 7.5). The eluted protein was further purified using size exclusion chromatography with a Superdex^TM^ 75 10/300 GL column (Cytiva, Uppsala, Sweden) in PBS buffer (pH: 7.5). The gene of M2e-Nb_C55A_C112.1S_ was recombined into pET28a vector (Novagen, Beijing, China) and transformed into SHuffle T7 Express Competent *E. coli* (NEB, Ipswich, MA, USA). The cells were cultured in 2YT medium supplemented with 50 mg/L kanamycin at 37 °C, and when the OD_600_ reached 0.6, the M2e-Nb_C55A_C112.1S_ was induced for expression with 0.1 g/L IPTG for 18 h at 16 °C. The purification steps were the same as those for the aforementioned antigen GFP-BC2-tag.

### 4.5. Detection and Quantification of Free Sulfhydryls in a Single-Domain Antibody Using Mass Spectrometry

The lyophilized lyso-VNAR was dissolved to 100 µL with a final concentration of 100 μM by adding ultra-pure water (18.2 MΩ/cm). Then, 90 µL of the protein sample was taken out and reduced with 10 µL of 50 mM of dithiothreitol (DTT) at room temperature for 30 min. The reduced protein sample was further alkylated with 100 mM of iodoacetamide (IAM) for 1 h in the dark at a ratio of 3 µL per 50 µg of protein. IAM could react with the free thiols of cysteine and prevent disulfide bond formation. Excess DTT and IAM were removed by 3 kDa centrifuge filters. The sample was diluted to 10 µM using acidified acetonitrile-water and then loaded into a nano-electrospray ionization MS system. Mass spectrometry samples were acquired on an Orbitrap Fusion Lumos Tribrid mass spectrometer (Thermo Scientific, Waltham, MA, USA). Data analysis and protein simulations were carried out on an XCalibur Qual Browser v4.3.

### 4.6. Affinity Measurements with SPR

SPR experiments were performed using a Biacore 8k instrument (Cytiva, Uppsala, Sweden). The lysozyme and GFP-BC2-tag were diluted to 20 µg/mL in a buffer containing 10 mM sodium acetate (pH: 4.5) and then immobilized onto a CM5 chip via amine groups using an amine coupling kit (Cytiva, Uppsala, Sweden). Different concentrations of samples (lyso-VNAR and reduced-state lyso-VNAR with concentrations ranging from 156 nM to 2500 nM and lyso-VNAR mutants, BC2-Nb, reduced-state BC2-Nb, BC2-Nb mutant with concentrations ranging from 312.5 nM to 5000 nM) were then applied to the chip surface at a flow rate of 30 µL/min. For the reduced-state sample, the lyso-VNAR and BC2-Nb were reduced with 5 mM DTT for 30 min before loading. The experiments for the WT and mutants were conducted with a running buffer containing 137 mM NaCl, 2.7 mM KCl, 10 mM Na_2_HPO_4_, and 1.8 mM KH_2_PO_4_ (pH: 7.5) at room temperature, and experiments for the reduced-state sample were carried out with the same condition, except for the running buffer containing 5 mM DTT. The K_D_ was determined using 1:1 Langmuir-binding model analysis, as provided by Biacore Insight Evaluation 3.0.12 software (Cytiva, Uppsala, Sweden). The SPR experiments used SEC-purified monomeric samples which had been identified using SDS-PAGE. The concentrations of the protein samples were estimated by measuring UV absorbance at 280 nm with an NP80 NanoPhotometer (Implen, Munchen, Germany).

### 4.7. Co-Purification by SEC

Antigens and single-domain antibodies with a concentration of 30 μM were incubated at 4 °C for 1 h with a 1:1 molar ratio. The lyso-VNAR, BC2-Nb, B8-VNAR, and M2e-Nb were reduced with 5 mM DTT for 30 min before incubation for the reduced-state sample. The sample was further purified by size exclusion chromatography using a Superdex^TM^ 75 10/300 GL column (Cytiva, Uppsala, Sweden) in PBS buffer (pH: 7.5) with or without 5 mM DTT for the reduced-state sample and other samples. The elution fractions were collected and analyzed with SDS-PAGE.

### 4.8. Thermal Stability Measurement

Single-domain antibodies with a concentration of 1 mg/mL were loaded into the Uncle system (Unchained Labs, Sunnyvale, CA, USA) to measure their intrinsic fluorescence. The excitation wavelength was set to 266 nm, and the emission spectra were recorded from 250 nm to 720 nm. The single-domain antibodies were reduced with 5 mM DTT prior to loading for the reduced-state sample. The thermal stability of different single-domain antibodies was evaluated through the heat-induced unfolding process, which was calculated from the barycentric mean of the intrinsic fluorescence emission spectra collected from 25 to 95 °C using a thermal ramp of 1 °C per minute in PBS buffer (pH: 7.5). The thermal unfolding experiments were analyzed using Uncle Analysis v6.01 Software (Unchained Labs, Sunnyvale, CA, USA). Each measurement was conducted in triplicate.

### 4.9. Molecular Dynamics (MD) Simulations

The initial conformations for the wild-type BC2-Nb and lyso-VNAR were down-loaded from PDB (PDB ID: 5IVN and 2I26, respectively) [41]. The structure prediction of non-canonical cysteine mutants was performed using AlphaFold 3 [42] and Modeller v9.25 [43]. These structures were similar based on PyMOL’s [44] alignment results (Figure S6A,B). Modeller’s predicted structures were chosen for the following simulations.

All of the systems were set up using VMD v1.9.3 software [45]. Firstly, all systems were solvated in a rectangular box filled with water molecules, with an 18.0 Å buffer distance from the margins to any solute atom. The charged amino acids were protonated automatically while assuming a constant pH of 7.0. The ion concentration of 0.20 M and electroneutrality were reached by adding counterions (Na^+^ and Cl^−^). The CHARMM36 all-atom protein force field and TIP3P model were used to parameterize the single-domain antibody and water molecules [46].

For the MD simulation processes, firstly, every system was optimized using a gradual process involving 500 step minimization and a 50 ns MD simulation with different restraining constants (i.e., 10.0, 5.0, 2.5, 1.25, and 0.25 kcal/(mol·Å^2^)). Then, a 1600 ns MD simulation was carried out for every system, and the last 1000 ns trajectories were used for analysis. In this paper, all MD simulations were run using ACEMD v3.7.3 software with the default configure parameters, A Langevin thermostat was set to 298.15 K, and a Berendsen barostat was set to 1 atm [47]. Finally, all trajectories were analyzed using the Wordom program v0.22 [48]. Figures were generated using PyMOL v2.5.2 and ChimeraX v1.5 [49,50].

## Figures and Tables

**Table 1:** The melting temperature (Tm) values of the lyso-VNAR, reduced-state lyso-VNAR, and mutants (*n* = 3).

VNARs	Tm (°C) (Mean ± SD)
lyso-VNAR	56.4 ± 0.8
lyso-VNAR (5 mM DTT)	41.0 ± 0.3
lyso-VNAR_C30G_C112.1A_	42.1 ± 1.3
lyso-VNAR_C30L_C112.1L_	44.5 ± 0.7
lyso-VNAR_C30W_C112.1V_	47.7 ± 1.3
lyso-VNAR_C30A_C112.1S_	44.7 ± 1.4
lyso-VNAR_C30G_C112.1L_	42.5 ± 0.3
lyso-VNAR_C30L_C112.1A_	44.5 ± 0.3
lyso-VNAR_C30L_C112.1V_	44.7 ± 0.8
lyso-VNAR_C30P_C112.1T_	37.5 ± 0.4

**Table 2:** The melting temperature (Tm) values of the BC2-Nb, reduced-state BC2-Nb, and mutant (*n* = 3).

VHHs	Tm (°C) (Mean ± SD)
BC2-Nb	70.4 ± 0.8
BC2-Nb (5 mM DTT)	64.5 ± 1.0
BC2-Nb_C55A_C110S_	63.1 ± 1.2

## Data Availability

All data are provided in the article and the Supplementary Materials.

## Supplementary Materials

The following supporting information can be downloaded at https://www.mdpi.com/article/10.3390/ijms25189801/s1.
