# Supplementary material for: Functional Divergence in the Affinity and Stability of Non-Canonical Cysteines and Non-Canonical Disulfide Bonds: Insights from a VHH and VNAR Study
Source: Int J Mol Sci. 2024 Sep 11;25(18):9801. doi: 10.3390/ijms25189801 (PMC11432006; doi:10.3390/ijms25189801)
Supplement: Supplementary file 1 [file ijms-25-09801-s001.zip › ijms-3163586-supplementary-final.pdf]

Supporting Information

Functional Divergence in the Affinity and Stability of Non-Canonical Cysteines and Non-Canonical Disulfide Bonds: Insights from a VHH and VNAR Study

Mingce Xu [1,2], Zheng Zhao [3,4], Penghui Deng [1,2], Mengsi Sun [5], Cookson K. C. Chiu [5], Yujie Wu [5], Hao Wang [1,6,*] and Yunchen Bi [1,2,6,*]

1 CAS and Shandong Province Key Laboratory of Experimental Marine Biology, Center for Ocean Mega-Science, Institute of Oceanology, Chinese Academy of Sciences, Qingdao 266071, China

2 University of Chinese Academy of Sciences, Beijing 100049, China

3 School of Data Science, University of Virginia, Charlottesville, VA 22904, USA

4 Department of Biomedical Engineering, University of Virginia, Charlottesville, VA 22904, USA

5 Shenzhen Bay Laboratory, Shenzhen 518055, China

6 Laboratory for Marine Biology and Biotechnology, Qingdao Marine Science and Technology Center, Qingdao 266071, China

* Correspondence : wanghao@qdio.ac.cn (H.W.); yunchenbi@qdio.ac.cn (Y.B.); Tel.: +86-0532-8289-8860 (H.W.); +86-0532-8289-3630 (Y.B.)

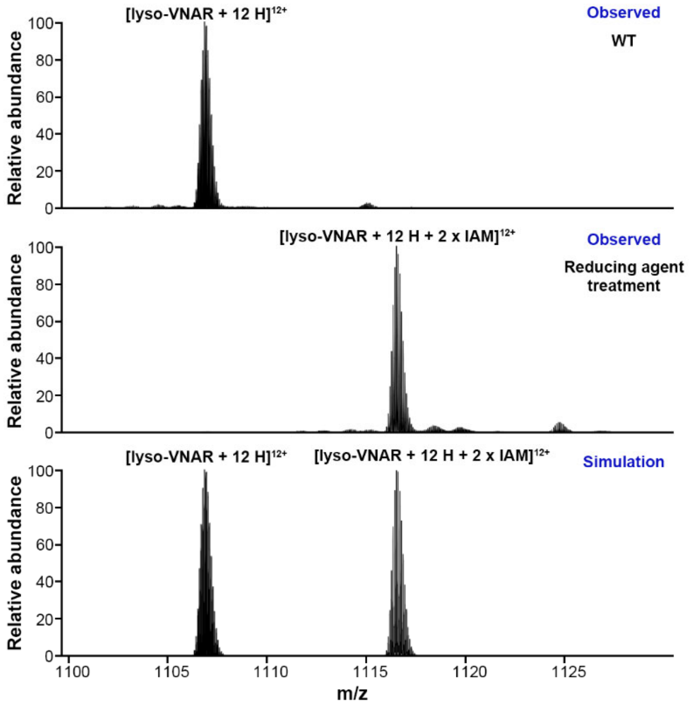

WT (unreacted)

Component	Relative Abundance
[lyso-VNAR + 12 H]$^{12+}$	100 %
[lyso-VNAR + 12 H + 2 x IAM]$^{12+}$	0 %

Reducing agent treatmet (reacted with DTT and IAM)

Component	Relative Abundance
[lyso-VNAR + 12 H]$^{12+}$	0 %
[lyso-VNAR + 12 H + 2 x IAM]$^{12+}$	100 %

Figure S1. The mass spectrometry data of lyso-VNAR. Nano-electrospray ionization mass spectra of lyso-VNAR (top), DTT treated sample (middle) and calculated non-canonical disulfide bond reduced and oxidized state sample (below), indicating non-canonical cysteines totally form the disulfide bond in lyso-VNAR sample. And one disulfide bond was reduced with DTT treatment as only 2 IAM were noticed from the MS spectra. This result indicates that the non-canonical disulfide bond of single-domain antibody was completely reduced after 5 mM DTT treatment, because all thiols of cysteines became free and accessible to react with IAM.

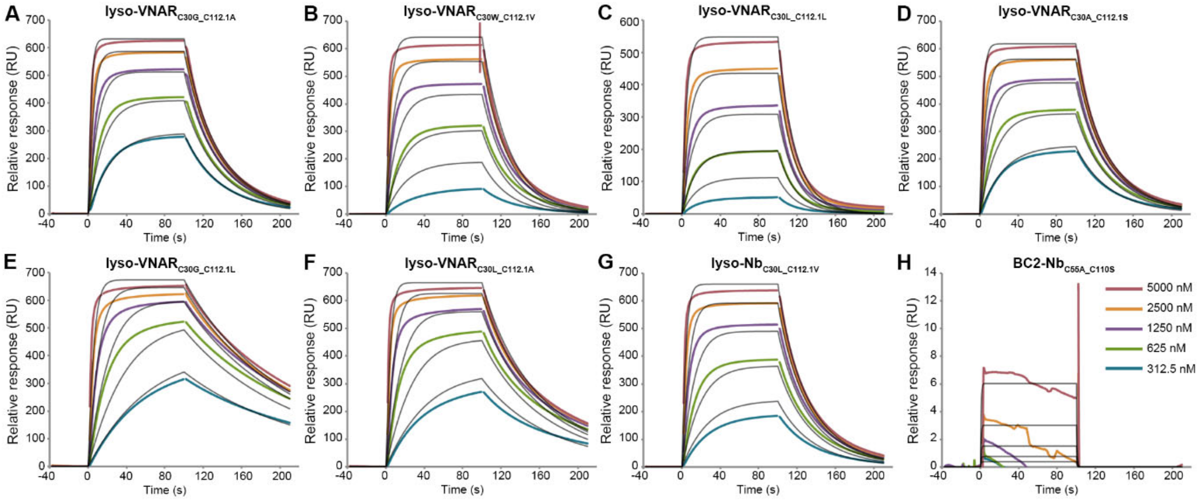

Figure S2. SPR-based equilibrium binding experiment between variants of lyso-VNAR, BC2-Nb and antigens.

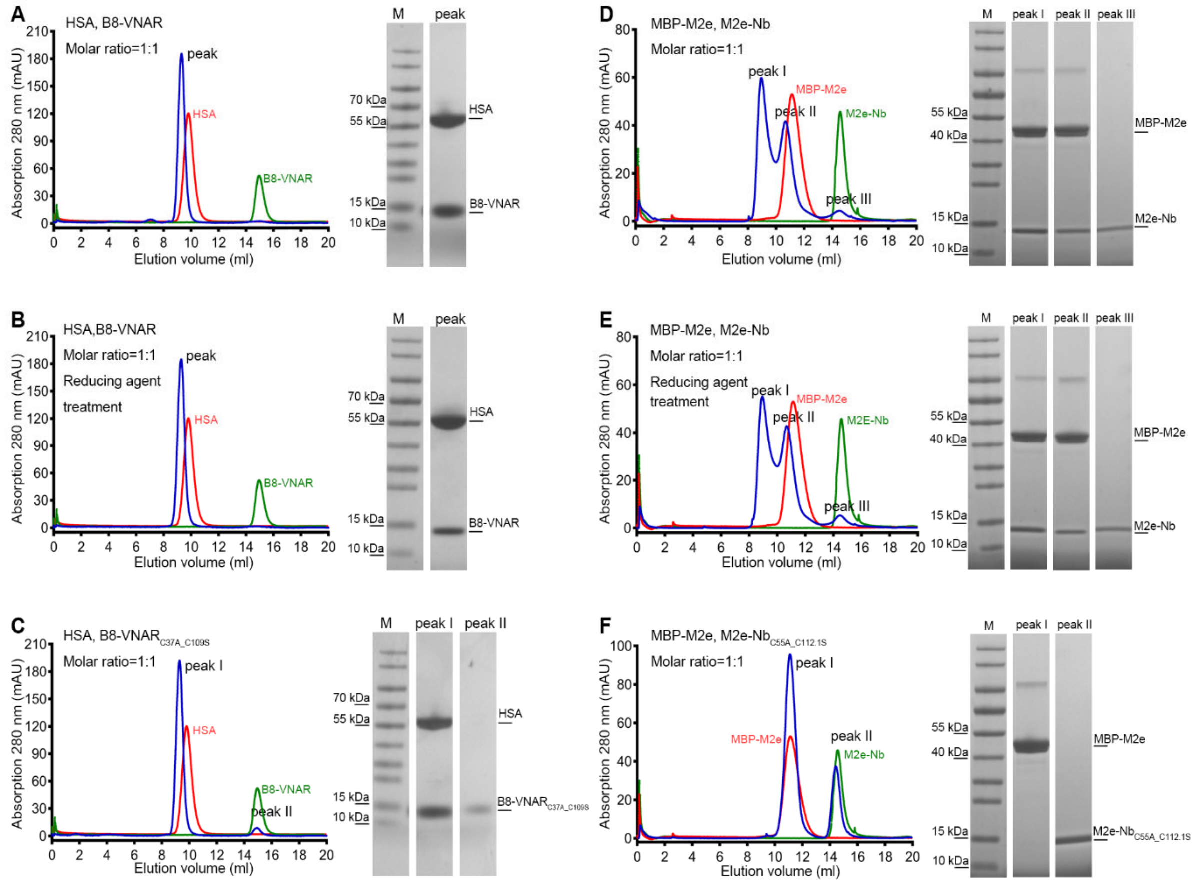

Figure S3. Co-purification SEC and SDS-PAGE analysis of the B8-VNAR binding with HSA, and M2e-Nb binding with MBP-M2e. B8-VNAR and M2e-Nb was incubated with the corresponding antigen HSA and MBP-M2e with 1:1 molar ratio respectively. The size exclusion chromatography was conducted followed by SDS-PAGE analysis to detect the single-domain antibody and antigen complex formation. Regardless of the disulfide bond state, the B8-VNAR were eluted together with HSA as a single peak at 9.3 mL from a Superdex^{TM} 75 10/300 GL column, which indicates B8-VNAR binds HSA tightly and form complex (A, B). The two peaks were observed from the B8-VNAR$_{C37A_C109S}$ variant, verified as complex and B8-VNAR sequentially. The appearance of additional B8-VNAR peak suggested the affinity loss due to the mutation of non-canonical cysteines (C). M2e-Nb could bind to MBP-M2e in different stoichiometric ratios. Either the WT or DTT treated samples, M2e-Nb and MBP-M2e could form two complex peaks (D, E). However, M2e-Nb$_{C55A_C112.1S}$ sample shows the complete loss of binding capacity with MBP-M2e. The two peaks observed were corresponded to MBP-M2e and M2e-Nb, respectively (F). The red and green curves represent individual SEC profiles of the antigen and sdAb, respectively, providing a clearer depiction of the shifts in elution positions of the complex peaks.

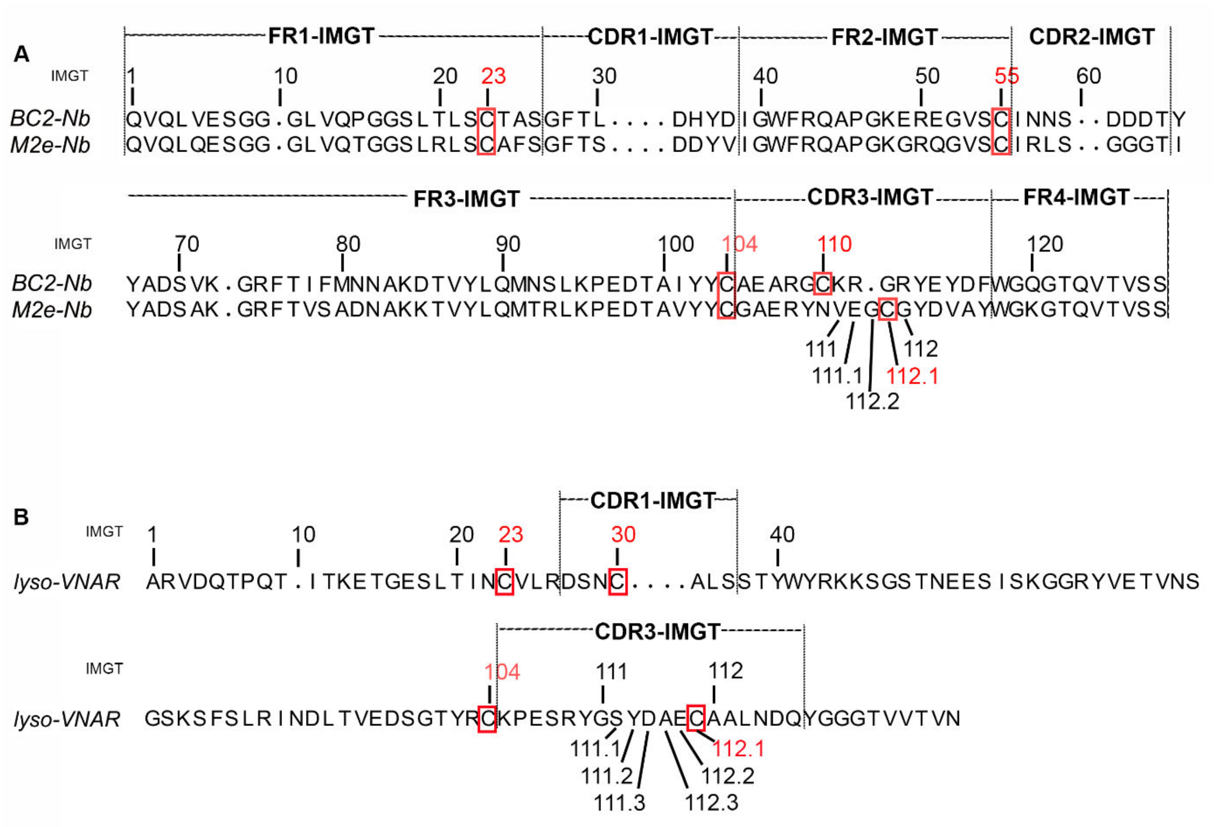

Figure S4. The amino acid sequences of BC2-Nb, M2e-Nb (A) and lyso-VNAR (B). The positons of amino acids were numbered using IMGT numbering system. All the cysteines were marked with red boxes. The FRs and CDRs of VHHs and CDRs of lyso-VNAR were delineated according to IMGT standardized delimitation.

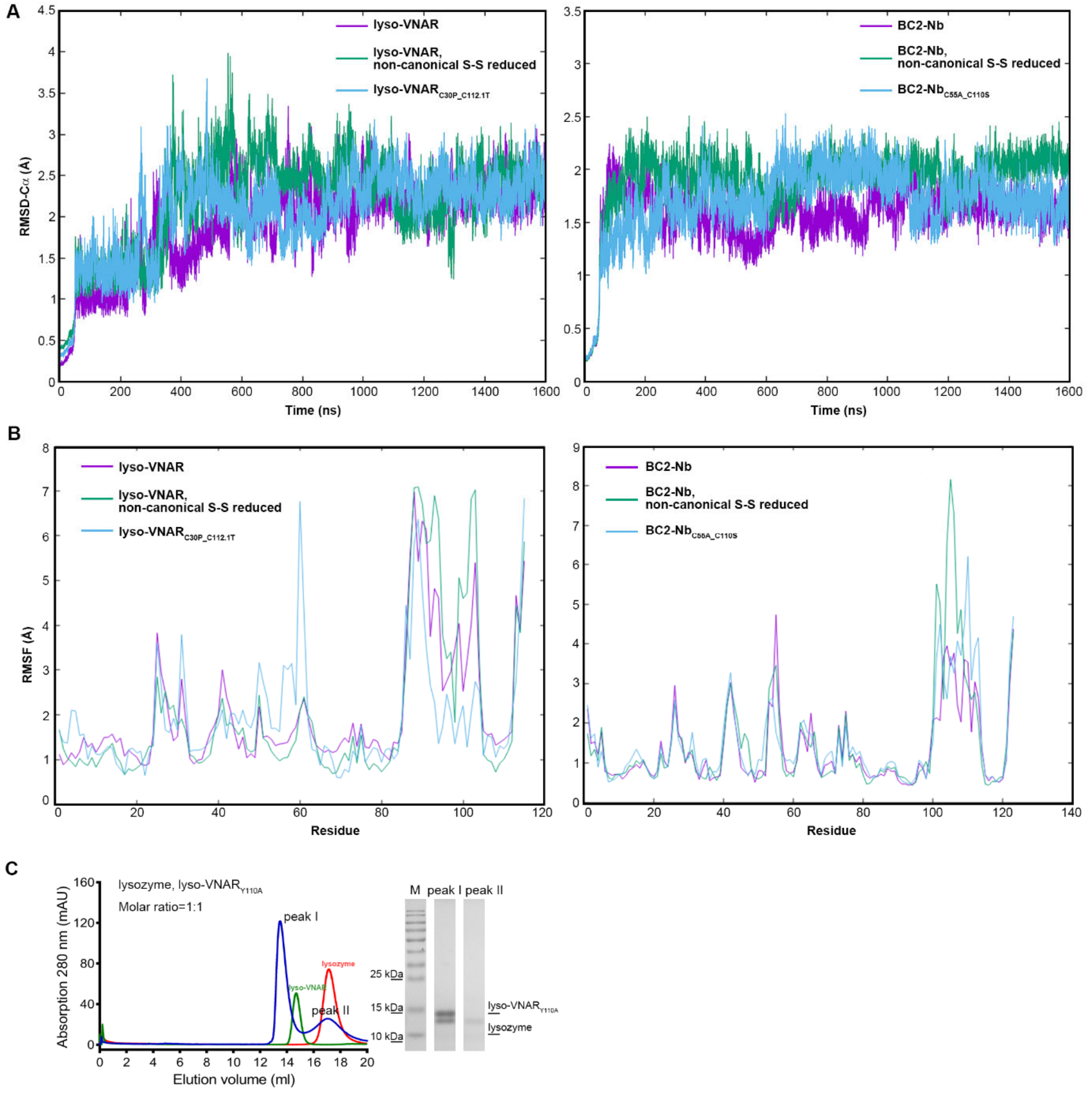

Figure S5. RMSD and RMSF calculated for Cα atoms of all residues in lyso-VNAR and BC2-Nb, and the importance of Tyr110 in lyso-VNAR affinity verified by lyso-VNAR$_{Y110A}$ variant. (A) The total root mean square deviation (RMSD) of the Cα in the 1600 ns molecular dynamics (MD) simulation for the lyso-VNAR (left, purple), reduced-state lyso-VNAR (left, green), lyso-VNAR$_{C30P_C112.1T}$ (left, blue), BC2-Nb (right, purple), reduced-state BC2-Nb (right, green), BC2-Nb$_{C55A_C110S}$ (right, blue). (B) RMSF plots of different lyso-VNAR and BC2-Nb systems: lyso-VNAR (left, purple), reduced-state lyso-VNAR (left, green), lyso-VNAR$_{C30P_C112.1T}$ (left, blue), BC2-Nb (right, purple), reduced-state BC2-Nb (right, green), BC2-Nb$_{C55A_C110S}$ (right, blue). (C) Size exclusion chromatography and SDS-PAGE analysis of the lyso-VNAR$_{Y110A}$ and antigen complex formation. There were two peaks eluted from the loading sample. The primary and secondary peak were diagnosed as the complex and lysozyme

by SDS-PAGE analysis, respectively. The red and green curves represent individual SEC profiles of the antigen and sdAb, respectively, providing a clearer depiction of the shifts in elution positions of the complex peak.

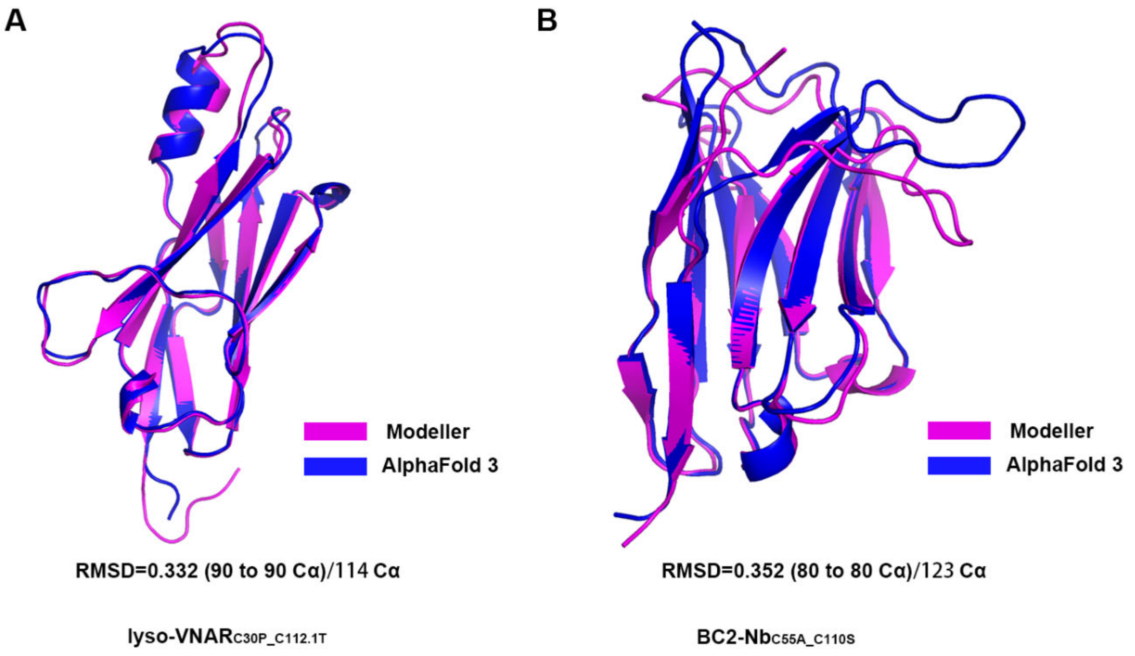

Figure S6. Alignment of the predicted mutant structures with Modeller and AlphaFold 3. The structures of mutant lyso-VNAR$_{C30P_C112.1T}$ and BC2-Nb$_{C55A_C110S}$ were predicted by AlphaFold 3 (blue) and Modeller (magenta). These structures were then aligned by PyMOL. The RMSD values calculated with Cα are 0.332 for the mutant lyso-VNAR$_{C30P_C112.1T}$ and 0.352 for the mutant BC2-Nb$_{C55A_C110S}$.

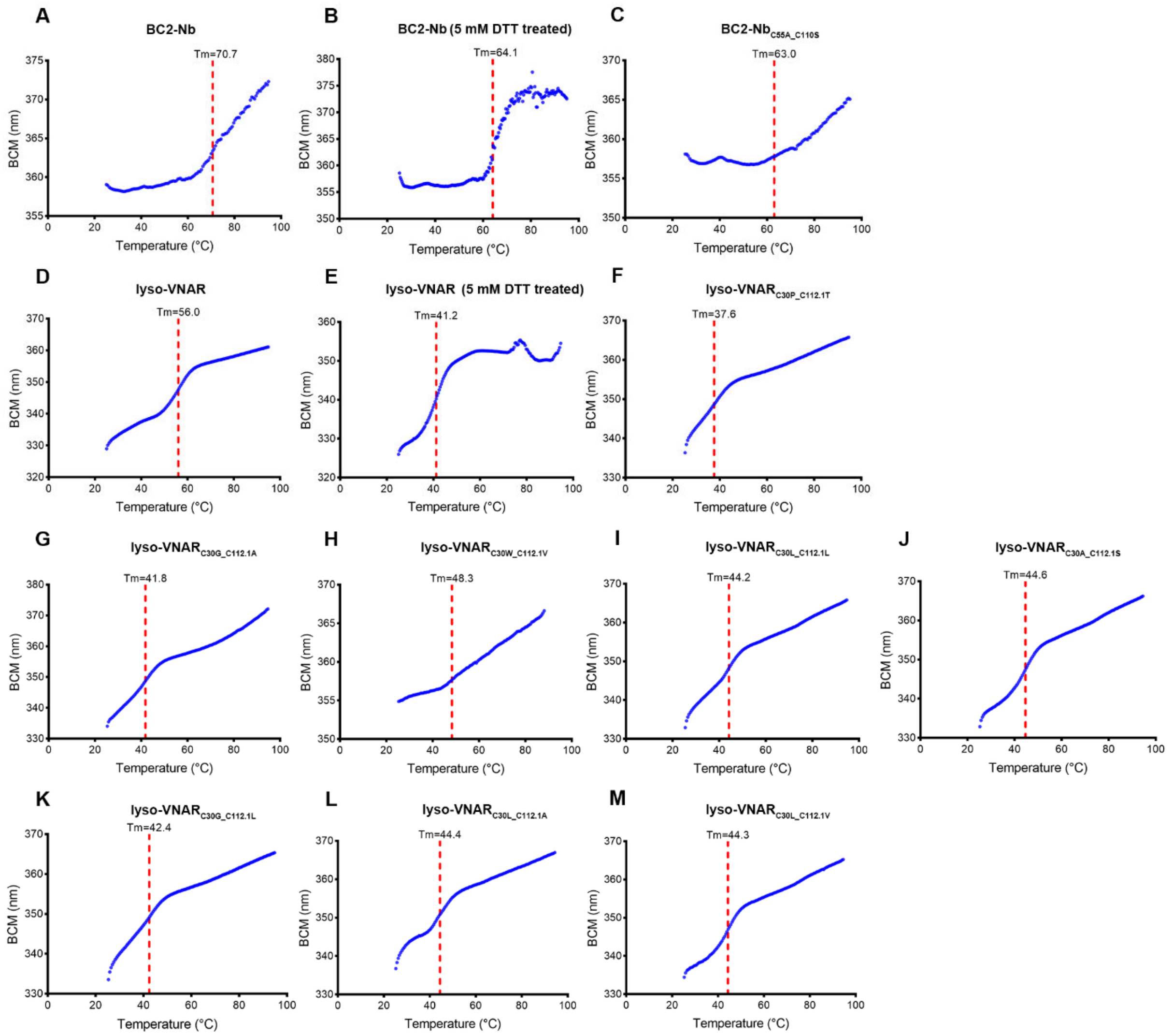

Figure S7. Representative melting temperature curves of single-domain antibodies.
